# Preliminary Investigation of the Biomarkers of Acute Renal Transplant Rejection Using Integrated Proteomics Studies, Gene Expression Omnibus Datasets, and RNA Sequencing

**DOI:** 10.3389/fmed.2022.905464

**Published:** 2022-05-12

**Authors:** Shuai Han, Wenjun Zhao, Cuili Wang, Yucheng Wang, Rong Song, Hermann Haller, Hong Jiang, Jianghua Chen

**Affiliations:** ^1^Kidney Disease Center, The First Affiliated Hospital, College of Medicine, Zhejiang University, Hangzhou, China; ^2^Key Laboratory of Nephropathy, Hangzhou, China; ^3^Institute of Nephropathy, Zhejiang University, Hangzhou, China; ^4^Zhejiang Clinical Research Center of Kidney and Urinary System Disease, Hangzhou, China; ^5^Department of Nephrology, Hannover Medical School, Hanover, Germany

**Keywords:** biomarkers, renal allograft rejection, proteomics studies, gene expression omnibus datasets, RNA sequencing

## Abstract

A kidney transplant is often the best treatment for end-stage renal disease. Although immunosuppressive therapy sharply reduces the occurrence of acute allograft rejection (AR), it remains the main cause of allograft dysfunction. We aimed to identify effective biomarkers for AR instead of invasive kidney transplant biopsy. We integrated the results of several proteomics studies related to AR and utilized public data sources. Gene ontology (GO) and pathway analyses were used to identify important biological processes and pathways. The performance of the identified proteins was validated using several public gene expression omnibus (GEO) datasets. Samples that performed well were selected for further validation through RNA sequencing of peripheral blood mononuclear cells of patients with AR (*n* = 16) and non-rejection (*n* = 19) from our medical center. A total of 25 differentially expressed proteins (DEPs) overlapped in proteomic studies of urine and blood samples. GO analysis showed that the DEPs were mainly involved in the immune system and blood coagulation. Pathway analysis showed that the complement and coagulation cascade pathways were well enriched. We found that immunoglobulin heavy constant alpha 1 (IGHA1) and immunoglobulin κ constant (IGKC) showed good performance in distinguishing AR from non-rejection groups validated with several GEO datasets. Through RNA sequencing, the combination of IGHA1, IGKC, glomerular filtration rate, and donor age showed good performance in the diagnosis of AR with ROC AUC 91.4% (95% CI: 82–100%). Our findings may contribute to the discovery of potential biomarkers for AR monitoring.

## Introduction

Renal transplantation is considered the best treatment for patients with end-stage renal disease (ESRD), which leads to a higher survival rate and a better quality of life (1). Although advances have been made in immunosuppressive drugs and surgical methods, the long-term outcomes of transplanted kidneys are not satisfactory. Patients with acute rejection (AR) after kidney transplantation are at an increased risk of developing chronic allografts and have reduced long-term graft survival (2).

Rapid advancements in genomics, transcriptomics, and proteomics technologies have promoted their application in understanding graft injury mechanisms. They have revealed promising biomarkers for reflecting the underlying biological processes (3). The genome is relatively unchanged and static; however, the proteome shows profound variation between different conditions and individuals. Proteomics is an effective technique for discovering biomarkers that can be used to understand the pathogenesis of various diseases and for non-invasive diagnoses. A large amount of data has been obtained from proteomics, with a deeper understanding of the pathophysiology of renal allograft rejection.

In recent years, many researchers have uploaded experimental data to public databases for validation or further use by other scientific researchers. Therefore, public databases have become an advantageous resource in disease research, especially for the discovery and verification of biomarkers. They have saved valuable time and resources for subsequent researchers. There are already some good examples showing that mining public databases is an effective and practical method for optimizing studies (4).

In this study, we acquired differentially expressed proteins (DEPs) in urine and blood samples from proteomic studies related to acute renal allograft rejection. Gene ontology (GO) and pathway analysis of the DEPs revealed that some biological processes were associated with the immune system and probably played important roles in acute allograft rejection. We then used public datasets to identify potential biomarkers for the diagnosis of AR and non-rejection groups. For further validation, we performed RNA sequencing of peripheral blood mononuclear cells (PBMCs) of patients with AR and in the non-rejection groups. The results showed that immunoglobulin κ constant (IGKC) region and immunoglobulin heavy constant alpha 1 (IGHA1) had good performance in distinguishing AR from non-rejection. The insights of this study may help deepen the understanding of the mechanism of acute allograft rejection and identify potential biomarkers for further characterization.

## Materials and Methods

### Acquisition of Differentially Expressed Proteins

We included 12 proteomic studies related to renal allograft rejection from 2011 to 2021, including six used urine samples and six used blood samples (Table 1). For further analysis, we integrated the results to identify DEPs between renal allograft rejection and non-rejection.

**TABLE 1:** Summary of the selected proteomics studies.

Authors	Sample	Type of rejection	Proteomics method	Criteria for DEPs
Nele K. et al.	Urine	ABMR	CE-MS and LC-MS/MS	FC > 1
Hee Y. et al.	Urine	ABMR	LC-MS/MS	FC ≥ 2
Inge M. et al.	Urine	ABMR	iTRAQ-LC-MS/MS	log_2_(FC) ≥ 0.6, *p* < 0.05
Meera S. et al.	Urine	AR, CAN	Protein Array	FC ≥ 2
Tara K. et al	Urine	AR, CAN, BKVN	iTRAQ-LC-MS/MS	FC > 2, *p* < 0.01
Tara K. et al	Urine	AR, CAN, BKVN	iTRAQ-LC-MS/MS	FC > 1.5, *p* < 0.01
Yue Z. et al.	Serum	AR	iTRAQ-LC-MS/MS	FC > 1.2, Q < 0.05
Tara K. et al	Serum	AR, CAN, BKVN	iTRAQ-LC-MS/MS	FC ≥ 2, *p* < 0.05
Marianne D. et al.	Serum	AMVR	Protein Array	FC > 1.2, *p* < 0.05
Juliana D. et al.	Plasma	AR	LC-MS/MS	Ratio > 1^#^
Meera S. et al.	Serum	AR, CAN	Protein Array	FC > 1.2, *p* < 0.05
Gabriela V. et al.	Plasma	AR	iTRAQ-MALDI-TOF/TOF	FC > 1.15, *p* < 0.05

*ABMR, antibody-mediated rejection; AR, acute rejection; BKVN, BK virus nephritis; CAN, chronic allograft nephropathy; AMVR, acute microvascular rejection; iTRAQ, isobaric Tags for Relative and Absolute Quantitation; LC-MS/MS, liquid chromatography-mass spectrometry/mass spectrometry; CE-MS, capillary electrophoresis-mass spectrometry; MALDI-TOF/TOF, matrix-assisted laser desorption ionization-time of flight/time of flight; FC, fold change. ^#^The concentration of proteins from the Rejection group versus control group.*

### Gene Ontology and Pathway Enrichment Analysis

Robust DAVID tools^1^ were applied to GO analysis and pathway analysis, which supply a significant set of functional annotations to investigators to better understand the biological significance of DEPs. The GO terms included cellular component (CC), molecular function (MF), and biological process (BP). Significant pathways and GO items were defined for an adjusted *p*-value of < 0.05.

### Acquisition of Candidate Biomarkers

Nephroseq^2^ is a robust database that integrates many publicly available kidney gene expression profiles. It provides researchers with gene expression data for data mining and visualization. Additionally, it uses different expression data for meta-analysis, a function that has been widely used in recent years (5). Hence, we uploaded the DEPs to Nephroseq for meta-analysis to reduce the inspection scale and discover potential biomarkers for acute renal allograft rejection. The search parameters were set as follows: *p*-value, 0.05; fold change, 1.5; and group, acute rejection. We then downloaded the search results for further analysis.

### Validation of Potential Biomarkers

Human microarray data were downloaded from the GEO database.^3^ Normalized bulk RNA-Seq expression data (FPKM) were used to calculate the expression of candidate biomarkers in human samples. Among different samples, the receiver operating characteristic (ROC) curve was applied to evaluate the performance of biomarkers in distinguishing AR from non-rejection groups.

### Validation of RNA Sequencing Experiments

Thirty-five ESRD patients who underwent kidney transplantation at the Kidney Disease Center of the First Affiliated Hospital of Zhejiang University were included. Patient demographics are shown in Table 2. PBMCs were isolated from the blood of 16 patients with biopsy-proven AR and 19 patients without rejection. For RNA sequencing, approximately 3 ml of peripheral blood was stored at –80°C. We used Trizol Reagent (Invitrogen) to extract total RNA (1,000 ng) and then used the NanoDrop 2000 spectrophotometer (Thermo Fisher Scientific, Waltham, MA) to measure the quantity and quality of the total RNA. Agilent 2100 Bioanalyzer (Agilent Technologies Inc., Santa Clara, CA, United States) was used to measure RNA integrity. The mRNA sequencing and raw RNA-seq data were processed as previously described. FPKM was used to calculate the expression of the candidate biomarkers in the PBMCs of the patients, and ROC analysis was applied to evaluate the performance of biomarkers in distinguishing AR from non-rejection groups among different samples. This study was approved by the Institutional Review Board of Zhejiang University School of Medicine. The patients provided written informed consent to participate in the study.

**TABLE 2:** Demographic and clinical characteristics of kidney allograft recipients.

Characteristics	AR (*n* = 16)	Non-rejection (*n* = 19)	*P-*value
Recipient age (≤50 years, %)	50	57.9	0.74
Recipient sex (Male, %)	68.8	63.2	1
Donor age (≤50 years, %)	62.5	26.3	0.04*
Donor sex (Male, %)	56.2	47.4	0.74
CIT (min)	319.13 ± 214.32	213.79 ± 166.65	0.11
BUN (mmol/L)	19.64 ± 6.94	16.31 ± 5.57	0.13
SCR (μmol/L)	817.06 ± 297.3	701.26 ± 296.7	0.26
GFR (mL/min/1.73 m^2^)	6.78 ± 2.35	9.47 ± 4.03	0.02*
24-UPRO (g/L)	2.87 ± 1.73	2.41 ± 0.99	0.37
UA (μmol/L)	388.13 ± 121.1	351.74 ± 102.23	0.34
Induction type, n (%)			
Basiliximab	11	15	0.70
Thymoglobuline	5	4	
Primary kidney disease, n (%)			
Glomerulonephritis	10	16	0.147
Hypertension	3	0	
Polycystic kidney disease	1	0	
Others	2	3	

Numbers are presented as mean ± SD or count (percentage %). AR, acute rejection; CIT, cold ischemia time; BUN, blood urea nitrogen; SCR, serum creatinine; GFR, glomerular filtration rate; 24-UPRO, 24-h urine protein quantification; UA, uric acid.*Represents a statistical significance.

## Results

### Identification of Differentially Expressed Proteins

After integrating the reported DEPs between AR and non-rejection samples, 158 DEPs and 119 DEPs were identified from the blood and urine samples, respectively. A total of 25 DEPs overlapped between the two groups (Table 3). To identify the important DEPs more accurately, we excluded proteins with high intensity in blood and urine samples, including albumin, transferrin, alpha-1 acid glycoprotein, complement, immunoglobulin, fibrinogen, ceruloplasmin, alpha-2-macroglobulin, alpha-1-antitrypsin, apolipoprotein, plasminogen, haptoglobin, and prealbumin. Finally, the 25 DEPs that overlapped between the blood and urine samples were identified by proteomic analysis. We thought that these 25 DEPs had significant implications in renal allograft rejection, and they were further analyzed.

**TABLE 3:** Information of 25 DEPs.

Entry	Gene	Protein names	Protein families
P02671	FGA	Fibrinogen alpha chain	NS
P01834	IGKC	Immunoglobulin kappa constant	NS
P61769	B2M	Beta-2-microglobulin	Beta-2-microglobulin family
P68871	HBB	Hemoglobin subunit beta	Globin family
P02790	HPX	Hemopexin	Hemopexin family
P01877	IGHA2	Immunoglobulin heavy constant alpha 2	NS
P05154	SERPINA5	Plasma serine protease inhibitor	Serpin family
P04196	HRG	Histidine-rich glycoprotein	NS
P20742	PZP	Pregnancy zone protein	Aalpha-2-macroglobulin family
P04217	A1BG	Alpha-1B-glycoprotein	NS
P43652	AFM	Afamin	ALB/AFP/VDB family
P06727	APOA4	Apolipoprotein A-IV	Apolipoprotein A1/A4/E family
P01876	IGHA1	Immunoglobulin heavy constant alpha 1	NS
P01861	IGHG4	Immunoglobulin heavy constant gamma 4	NS
P02750	LRG1	Leucine-rich alpha-2-glycoprotein	NS
P01008	SERPINC1	Antithrombin-III	Serpin family
Q99598	TSNAX	Translin-associated protein X	Translin family
P00751	CFB	Complement factor B	Peptidase S1 family
P08311	CTSG	Cathepsin G	Peptidase S1 family
P62805	H4C1	Histone H4	Histone H4 family
P02679	FGG	Fibrinogen gamma chain	NS
P02675	FGB	Fibrinogen beta chain	NS
P16949	STMN1	Stathmin	Stathmin family
P51884	LUM	Lumican	SLRP family
P01019	AGT	Angiotensinogen	Serpin family

*According to Uniprot. NS, no data; SLRP, small leucine-rich proteoglycan.*

### Gene Ontology and Pathway Enrichment Analysis

**FIGURE 1:**
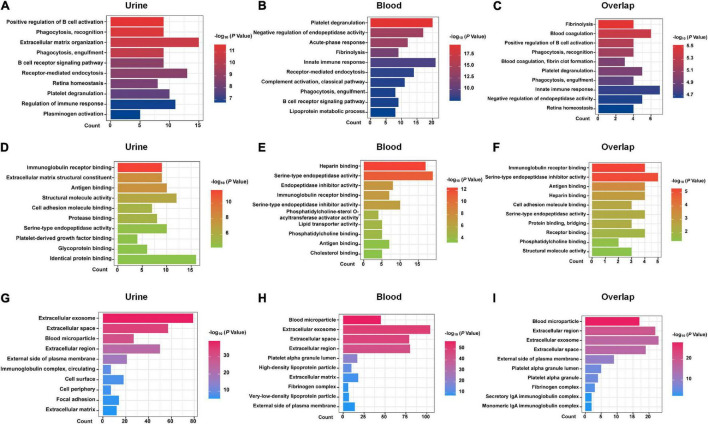
GO analysis with DAVID. GO analysis of the DEPs of urine samples, blood samples, and 25 shared DEPs based on proteomics studies are shown. **(A–C)** biological process (BP) items. **(D–F)** molecular function (MF) items. **(G–I)** cellular component (CC) items.

DEPs from urine and blood samples were further analyzed using DAVID tools. As shown in Figure 1, GO terms, including BP (Figures 1A–C), MF (Figures 1D–F), and CC (Figures 1G–I) were analyzed in detail. Several important BP items were enriched in urinary DEPs, including positive regulation of B-cell activation, phagocytosis, the B-cell receptor signaling pathway, regulation of immune response, and platelet degranulation. Similarly, some important BP items were enriched in blood samples, including platelet degranulation, acute-phase response, innate immune response, complement activation, phagocytosis, and the B cell receptor signaling pathway. The overlapping DEPs also showed many important BP items, including platelet degranulation, innate immune response, phagocytosis, and blood coagulation. In summary, most GO items were involved in the immune system and blood coagulation, suggesting that they probably play an important role in the process of acute allograft rejection.

**FIGURE 2:**
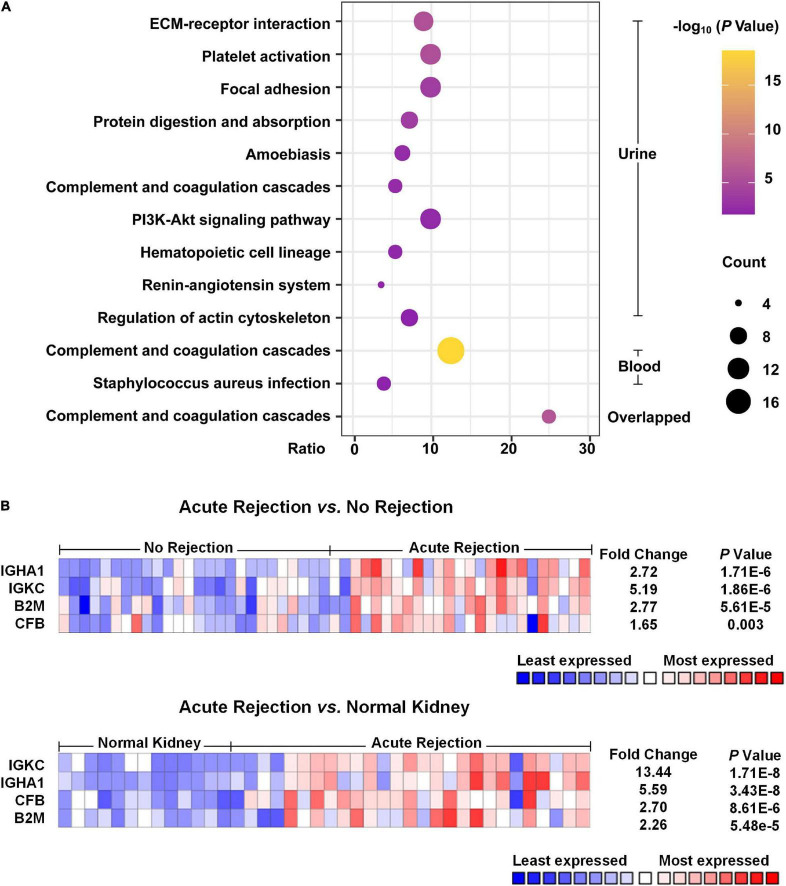
Pathway enrichment analysis and Nephroseq usage. **(A)** Pathway enrichment analysis of the DEPs was performed with DAVID. The top 10 pathways of urinary DEPs are shown. As for analysis of the DEPs from blood samples, only two pathways were identified with an adjusted *p*-value < 0.05. **(B)** Heat map of the expression of potential biomarkers based on Nephroseq. Compared with non-rejection and normal kidney tissue groups, acute rejection showed that IGKC, IGHA1, B2M and CFB were significantly different with a *p*-value < 0.05 and fold change > 1.5.

As shown in Figure 2A, the pathway analysis of the DEPs detected from blood samples was not well enriched, and only two items were identified, including complement and coagulation cascades and *Staphylococcus aureus* infection. We found that the complement and coagulation cascade pathways were enriched both in the DEPs of urine samples and blood samples, as well as in the overlapped DEPs, suggesting that this pathway probably played an important role in the occurrence of acute allograft rejection.

### Potential Biomarkers Identified From Differentially Expressed Proteins

**FIGURE 3:**
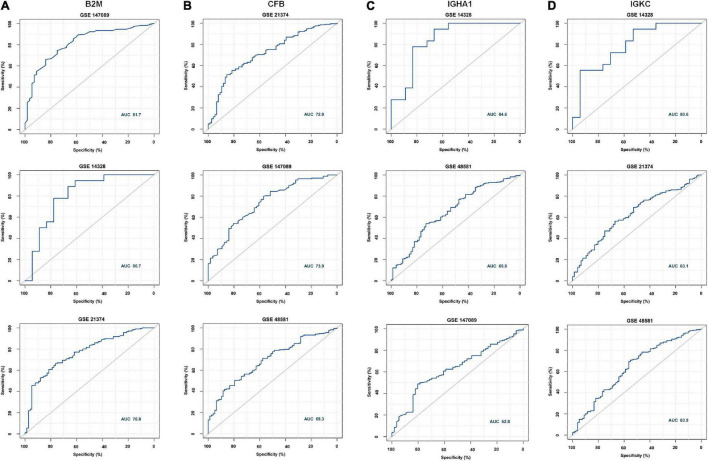
Receiver operating characteristic (ROC) curve of B2M, CFB, IGHA1, and IGKC in different GEO data sets. **(A)** ROC of B2M in GSE147089, GSE14328, and GSE21374 with AUC values of 81.7, 80.7, and 76.8, respectively. **(B)** ROC of CFB in GSE21374, GSE147089, and GSE48581 with AUC values of 72, 73.9, and 69.3, respectively. **(C)** ROC of IGHA1 in GSE14328 and GSE147089 with AUC values of 84.5, 65.8, and 62.8, respectively. **(D)** ROC of IGKC in GSE14328, GSE21374, and GSE48581 with AUC values of 80.6, 63.1, and 63.9, respectively.

**FIGURE 4:**
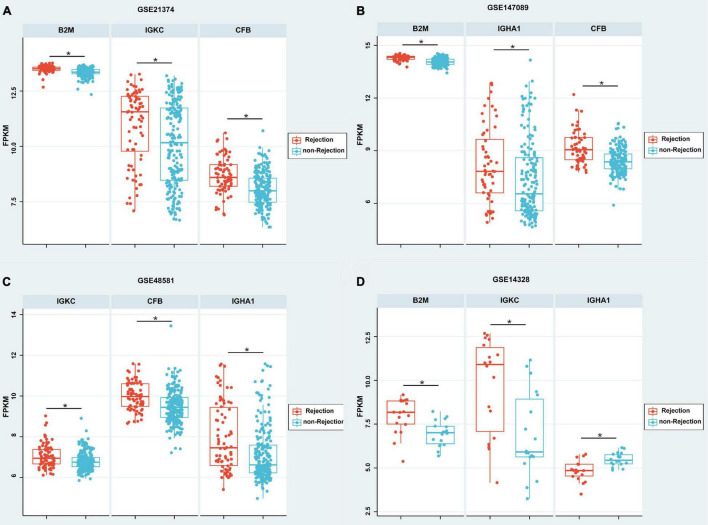
Boxplot of the expressions of B2M, CFB, IGHA1, and IGKC in different GEO data sets. **(A–C)** According to GSE21374, GSE147089, and GSE48581, the expression of B2M, IGHA1, IGKC, and CFB were significantly higher in the rejection group than in the non-rejection group. **(D)** According to GSE21374, the expressions of B2M and IGKC were significantly higher; however, IGHA1 was significantly lower in the rejection group than in the non-rejection group. Each point represents a patient. *p < 0.05.

According to the meta-analysis of Nephroseq, 25 DEPs were uploaded to the website to determine whether they were differentially expressed in patients with AR. As shown in Figure 2B, beta-2-microglobulin (B2M), complement factor B (CFB), IGHA1, and IGKC showed significant differences between patients with AR and non-rejection patients based on the Sarwal Transplant Kidney Dataset (6) with a *p*-value < 0.05 and a fold change > 1.5. Similar results were also observed in patients with AR and with normal kidney tissues. Since IGHA1, IGKC, B2M, and CFB play important roles in the immune system, we further validated the four biomarkers in other public datasets. As shown in Table 4, we examined four publicly available transcriptome datasets (GSE14328, GSE21374, GSE48581, and GSE147089) to test the performance of these four biomarkers in distinguishing AR from non-rejection groups (7–10). As depicted in Figure 3, the four biomarkers showed relatively good performance (most AUC areas were larger than 0.7), suggesting their potential as efficient biomarkers. We also described the expression levels of the four biomarkers in the different datasets using boxplots. As shown in Figure 4, the expression of most biomarkers in the AR group was significantly higher than that in the non-rejection group.

**TABLE 4:** Summary of selected GEO data sets.

Accession	Organism	Sample size	Experiment type	Sample source
GSE14328	Human	Total *n* = 36; AR (*n* = 18) vs. STA (*n* = 18)	Expression profiling by array	Renal allograft biopsy
GSE21374	Human	Total *n* = 282; Rejection (*n* = 76) vs. non-rejection (*n* = 206)	Expression profiling by array	Renal allograft biopsy
GSE48581	Human	Total *n* = 300; Rejection (*n* = 78) vs. non-rejection (*n* = 222)	Expression profiling by array	Renal allograft biopsy
GSE147089	Human	Total *n* = 226; Rejection (*n* = 58) vs. non-rejection (*n* = 168)	Expression profiling by array	Renal allograft biopsy

*AR, acute allograft rejection; STA, allograft with stable renal function.*

### Potential Biomarkers Validated by RNA Sequencing

**FIGURE 5:**
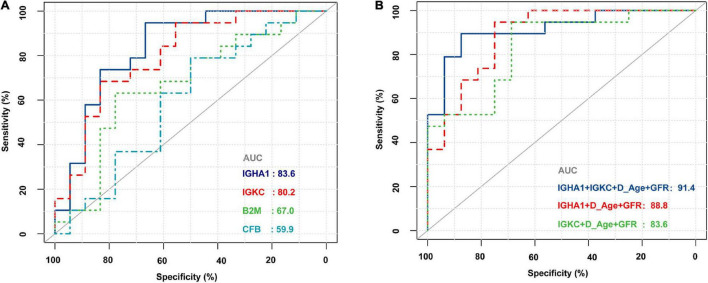
Receiver operating characteristic (ROC) curves of B2M, CFB, IGHA1, and IGKC based on RNA sequencing in PBMCs of renal allograft transplant patients. **(A)** ROC of B2M, CFB, IGHA1, and IGKC merely based on the expression of RNA-Seq. **(B)** ROC of different combinations of IGHA1, IGKC, GFR, and donor age. D_Age represents the donor age. IGHA1 + IGKC + D_Age + GFR AUC 91.4% (95% CI: 82–100%). IGHA1 + D_Age + GFR AUC 88.8% (95% CI: 77.8–99.9%). IGKC + D_Age + GFR AUC 83.6% (95% CI: 70.1–97%).

The demographic and clinical characteristics of the patients are presented in Table 2. The donor age and glomerular filtration rate (GFR) were significantly different between AR recipients and non-rejection recipients. There were no statistically significant differences between the two groups in terms of recipient age/sex, donor sex, cold ischemia time, blood urea nitrogen, serum creatinine, 24-h urine protein quantification, and uric acid levels. The four biomarkers were also detected by RNA-Seq, and we then used ROC analysis to evaluate their individual performance in distinguishing AR from the non-rejection group. As shown in Figure 5A, IGHA1 and IGKC showed relatively good performance, with AUCs of 83.6 and 80.2, respectively. Subsequently, we combined the four features, including donor age, GFR, IGHA1, and IGKC, to determine their performance in distinguishing AR from non-rejection. As shown in Figure 5B, the ROC of the four-index combination showed good performance with an ROC AUC of 91.4% (95% CI: 82–100%), suggesting its potential clinical use in monitoring transplant patients.

### Statistical Analyses

The results are presented as the means ± SD for continuous variables. Categorical variables were expressed in terms of rate (%) or composition ratio (%). For statistical comparisons of the clinical data between two groups, we used unpaired, two-tailed *t*-tests and chi-squared test. *P*-values < 0.05 were considered significant. All data were statistically analyzed using SPSS Statistics V20 (IBM Analytics), and statistical charts were created using GraphPad Prism software (version 8.0.1; GraphPad Software, San Diego, CA).

## Discussion

In recent years, the effective use of immunosuppressive drugs has sharply reduced the rate of renal graft rejection; however, AR still occurs in approximately 10–20% of total reported cases. It has been reported that every episode of rejection is closely associated with a poor graft survival rate, even after immunosuppressive treatment (11, 12). Therefore, it is necessary to further understand acute renal allograft rejection. Traditional non-invasive graft functional parameters for monitoring rejection, include glomerular filtration rate, serum creatinine levels, measurement of donor-specific human leukocyte antigen antibodies, proteinuria, lack sensitivity and specificity. Unmet clinical needs include non-invasive biomarkers with excellent sensitivity and specificity for kidney allograft rejection processes (13).

In the past decade, proteomics has been widely used in scientific research, especially for the discovery of effective biomarkers for various diseases. Many biomarkers detected by proteomic methods have shown good accuracy and sensitivity in the diagnosis of various types of rejection (14–16); however, there are some prominent flaws that limit their application. The results of proteomic studies often show significant differences among different researchers, laboratories, and methodologies. This condition is also reflected in the present study to some extent (Table 1). Therefore, we sought to identify meaningful proteins by reviewing proteomic studies related to acute allograft rejection, with the aim of reducing the variance among different studies. After excluding proteins with high abundance in body fluid, a total of 25 DEPs were shared between urine and blood samples. DEPs were considered to be potential markers and play an important role in acute allograft rejection.

Through GO and pathway analyses, we found common features shared by urine and blood samples, including B cell activation, phagocytosis, immune response, and platelet degranulation. These biological processes are involved in the immune system and play an important role in acute allograft rejection. It is well known that the immune system is closely related to AR, which can occur any time after allograft transplantation, including antibody-mediated rejection and acute T cell-mediated rejection. Both of these rejections include the detected biological processes. As for pathway analysis, complement and coagulation cascade pathways were found to be enriched in DEPs from urine and blood samples, suggesting that it is a key pathway that mediates the process of acute allograft rejection.

In our analysis, we found four proteins that were significantly increased in the AR groups compared to both non-rejection groups and normal kidney tissues (Figure 2B). To validate their performance in diagnosing AR, we used several GEO datasets published in the public. We found that the expression of B2M, CFB, IGHA1, and IGKC was very similar in different datasets, even though the absolute expression was different.

IGKC is an important component of immunoglobulins, which are involved in innate and adaptive immunity, and is an effective prognostic biomarker for breast cancer and non-small cell lung carcinoma (NSCLC) (17). Schmidt et al. reported that IGKC is a novel diagnostic marker for risk stratification in NSCLC and supported concepts to exploit the humoral immune response for anticancer therapy, which could be validated by transcriptomics and immunostaining at the protein level (17).

IGHA1 is an adaptive immune effector that is a highly abundant circulating protein with relatively stable blood concentrations (18). It was found to be differentially expressed in several diseases, such as chronic kidney disease, glioblastoma, hyperthyroidism, breast cancer, NSCLC, clear cell renal cell carcinoma, and autoimmune disease (18–23). Chitnis et al. reported a novel functional role of the human leukocyte antigen-B (HLA-B) locus, mediated by its intron-encoded miR-6891-5p. They identified a conserved miR-6891-5p target site in both IGHA1 and IGHA2 transcripts, suggesting that this miRNA modulates the expression of IGHA1 and IGHA2 (19). Since allograft rejection is usually associated with HLA, we considered that the role of miR-6891-5p and IGHA1 in allograft rejection mediated by HLA should be investigated further. We noticed that studies related to IGHA1 were based on proteomic methods, such as SWATH mass spectrometry, multiple reaction monitoring mass spectrometry assays, and matrix-assisted laser desorption/ionization time-of-flight mass spectrometry. Interestingly, several studies have reported that a combination of biomarkers, including IGKC and IGHA1, has good prognostic value in clear cell renal cell carcinoma and autoimmune diseases (24, 25). Similarly, our study showed that the combination of biomarkers, including IGKC and IGHA1, had potential prognostic value in acute renal allograft rejection.

As shown in Figure 3, most ROC had an AUC > 0.7. Hence, we hypothesized that these molecules are likely to have the potential to be effective biomarkers. For further validation, we performed RNA-Seq on PBMCs from patients whose clinical features and individual information were relatively abundant. As shown in Table 2, the clinical features of donor age and GFR were significantly different between the AR and non-AR groups. We also examined the performance of B2M, CFB, IGHA1, and IGKC in AR diagnosis using RNA-Seq data. IGHA1, IGKC, and B2M showed relatively good performances, especially for the previous two biomarkers (Figure 5A). Hence, we combined IGHA1, IGKC, clinical features of donor age, and GFR to evaluate their performance in the diagnosis of AR. The ROC of the four-index combination showed good performance with an ROC AUC of 91.4% (95% CI: 82–100%), suggesting its potential for clinical use in monitoring transplant patients, which should be validated further.

Our study had few limitations. First, in the process of identifying DEPs, we were unable to acquire whole raw data from the included studies for reanalysis, and the methods used to define DEPs were different among the studies, which might have influenced the results. Second, the sample size was relatively small, and more studies are needed to validate our findings. Third, recipients usually receive immunosuppressive treatments before transplantation, which may have an unaddressed effect on our results. Therefore, these limitations should be considered when testing the clinical utility of identified biomarkers in blinded prospective studies.

In summary, our study integrated the results of several proteomic studies and utilized public data sources to identify proteins of interest related to acute allograft rejection. Through bioinformatic analysis, we identified some important biological processes and pathways that probably play an important role in AR. Finally, we performed RNA-Seq of PBMCs obtained from patients in our study and found that the combination of IGHA1, IGKC, GFR, and donor age showed good performance with an ROC AUC of 91.4% (95% CI: 82–100%). An in-depth integrated analysis of proteomics and transcriptomic data revealed that IGHA1, IGKC, GFR, and donor age had potential for clinical use in monitoring transplant patients.

## Data Availability Statement

The data presented in the study are deposited in the China National Center for Bioinformation repository, accession number OMIX001115 (https://ngdc.cncb.ac.cn/omix/release/OMIX001115).

## Ethics Statement

This study was approved by the Institutional Review Board of the Zhejiang University School of Medicine. The patients/participants provided their written informed consent to participate in this study.

## Author Contributions

SH participated in the writing of the manuscript, performance of the research, and data analysis. WZ participated during the research with patients. CW participated in data analysis and in the writing of the manuscript. YW contributed in the research design. RS participated in data analysis. HH contributed analytic tools and data analysis. HJ contributed in the research design and in the writing of the manuscript. JC contributed in the research design and in the writing and revision of the manuscript. All authors contributed to the article and approved the submitted version.

## Conflict of Interest

The authors declare that the research was conducted in the absence of any commercial or financial relationships that could be construed as a potential conflict of interest.

## Publisher’s Note

All claims expressed in this article are solely those of the authors and do not necessarily represent those of their affiliated organizations, or those of the publisher, the editors and the reviewers. Any product that may be evaluated in this article, or claim that may be made by its manufacturer, is not guaranteed or endorsed by the publisher.

## Footnotes

1
https://david.ncifcrf.gov/home.jsp

2
https://www.nephroseq.org

3
https://www.ncbi.nlm.nih.gov/geo/
